# A Systems Level Analysis of Vasopressin-mediated Signaling Networks in Kidney Distal Convoluted Tubule Cells

**DOI:** 10.1038/srep12829

**Published:** 2015-08-04

**Authors:** Lei Cheng, Qi Wu, Marleen L. A. Kortenoeven, Trairak Pisitkun, Robert A. Fenton

**Affiliations:** 1 Center for Interactions of Proteins in Epithelial Transport, Department of Biomedicine, Aarhus University, 8000, Aarhus, Denmark; 2 Faculty of Medicine, Chulalongkorn University, Bangkok, Thailand

## Abstract

The kidney distal convoluted tubule (DCT) plays an essential role in maintaining body sodium balance and blood pressure. The major sodium reabsorption pathway in the DCT is the thiazide-sensitive NaCl cotransporter (NCC), whose functions can be modulated by the hormone vasopressin (VP) acting via uncharacterized signaling cascades. Here we use a systems biology approach centered on stable isotope labeling by amino acids in cell culture (SILAC) based quantitative phosphoproteomics of cultured mouse DCT cells to map global changes in protein phosphorylation upon acute treatment with a VP type II receptor agonist 1-desamino-8-D-arginine vasopressin (dDAVP). 6330 unique proteins, containing 12333 different phosphorylation sites were identified. 185 sites were altered in abundance following dDAVP. Basophilic motifs were preferential targets for upregulated sites upon dDAVP stimulation, whereas proline-directed motifs were prominent for downregulated sites. Kinase prediction indicated that dDAVP increased AGC and CAMK kinase families’ activities and decreased activity of CDK and MAPK families. Network analysis implicated phosphatidylinositol-4,5-bisphosphate 3-kinase or CAMKK dependent pathways in VP-mediated signaling; pharmacological inhibition of which significantly reduced dDAVP induced increases in phosphorylated NCC at an activating site. In conclusion, this study identifies unique VP signaling cascades in DCT cells that may be important for regulating blood pressure.

The peptide hormone arginine vasopressin (VP) is essential for maintaining extracellular fluid homeostasis. Although VP is often recognized for its water-retaining actions on the collecting duct (CD) via its effects on the water channel aquaporin-2 (AQP-2)^1^,^2^, VP also modulates sodium transport in the thick ascending limb (TAL), the distal convoluted tubule (DCT), the connecting tubule (CNT) and the collecting duct (CD) e.g.^3^,^4^,^5^. The effects of VP on the TAL, DCT and CD epithelial cells are likely via the VP type II receptor (V2R)^6^,^7^.

In the DCT, the molecular targets for VP include the epithelial Na channel ENaC (also regulated by VP in the CNT and CD) and the NaCl co-transporter NCC^8^. Acute V2R activation via VP increases ENaC activity by translocating ENaC to the apical plasma membrane, enhancing the stability of ENaC and increasing the channel open probability (reviewed extensively in^9^). Chronic activation of the V2R increases ENaC transcription and expression e.g.^10^. Chronic VP exposure also increases NCC abundance^8^, but acute VP treatment modulates NCC activity by directly increasing the transport activity of NCC already present in the plasma membrane, a process that is dependent on phosphorylation of conserved residues in the amino terminus of NCC^11^,^12^,^13^. Although VP actions to increase NCC phosphorylation and thus activation are modulated, at least in part, by the Ste20-like kinase, SPS-related proline/alanine-rich kinase (SPAK)^12^,^14^; whether VP induces phosphorylation and activates the upstream with-no-lysine serine/threonine kinases (WNKs), which are kinases known to regulate NCC activity is unknown.

Both ENaC and NCC play a critical role in the maintenance of blood pressure. This is highlighted by gain-of-function in ENaC and NCC underlying the hypertensive Liddle and Gordon’s syndrome^15^, and the role of both ENaC and NCC in the development of salt-sensitive hypertension^16^,^17^. As both ENaC and NCC are activated by VP, and under certain pathological conditions VP actions may increase sodium retention and subsequently blood pressure^18^, understanding of the complex signaling network that is activated in DCT cells following VP exposure is essential. In particular, identification of the signaling network activated by VP and accounts for this increased Na transport via NCC and/or ENaC is essential and may be relevant for uncovering new mechanisms important for regulating blood pressure.

As protein phosphorylation and dephosphorylation are dynamic protein modifications critical in virtually all cell signaling processes, we performed large-scale liquid chromatography tandem mass spectrometry (LC-MS/MS)-based phosphoproteomic profiling and quantification in mpkDCT cells, a model of the native DCT, to discover the key elements of VP-mediated signaling in the DCT. We provide a comprehensive signaling network that is activated by VP in DCT cells, and identify numerous new phosphorylation sites in key transporters and signaling molecules. Finally, pathway analysis studies identified phosphatidylinositol-4,5-bisphosphate 3-kinase (PI3K) and CAMKK dependent pathways as important signaling components of the VP type II receptor; pharmacological inhibition of these pathways *ex vivo* using low concentrations of selective inhibitors resulted in decreased NCC phosphorylation at an activating site.

## Results

### mpkDCT cells as a VP sensitive DCT model

**Figure 1:**
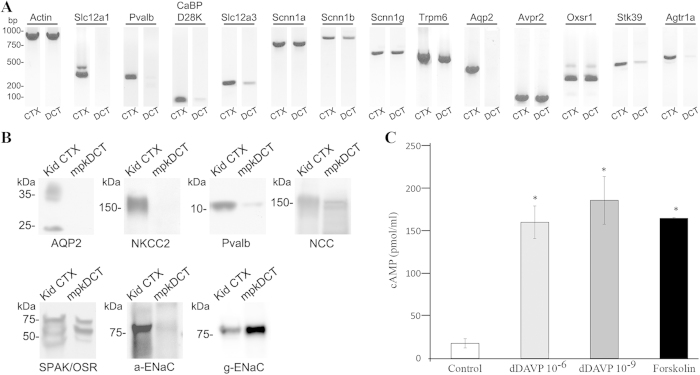
Characterization of mpkDCT cells. (**A**) RT-PCR and (**B**) Western blots demonstrate that mpkDCT cells express a similar cohort of molecules as native DCT cells. Mouse kidney cortex (CTX) was used as a positive control. (**C**) mpkDCT cells respond to acute dDAVP stimulation with significant increases in intracellular cAMP levels. Forskolin (25 uM) was used as positive control. * indicates p < 0.05 relative to control

To confirm that mpkDCT cells are a good model of the DCT, expression of various genes markers for the DCT, thick ascending limb (TAL) and connecting tubule (CNT) in these cells were determined. RT-PCR analysis (Fig. 1A) demonstrated expression of characteristic markers of native DCTs, including players in transcellular Ca^2+^ transport (*Pvalb, CaBPD28K, Trpm6*) and Na^+^ transport pathways (*Slc12a3, Scnn1a/b/g*), as well as key regulators of transcellular Na^+^ transport (*Avpr2, Agtr1a, Oxsr1, Stk39*). mpkDCT cells did not express markers of the TAL (*Slc12a1*) or the CNT (*Aqp2*). A number of these observations were confirmed at the protein level (Fig. 1B). The expression of ENaC (*Scnn1*) subunits at the mRNA and protein levels, coupled with the weak expression of parvalbumin (*Pvalb*) in mpkDCT cells is characteristic of the late DCT (DCT2). mpkDCT cells responded to 15 min dDAVP stimulation from their basolateral side with significant increases in intracellular cAMP levels (Fig. 1C). Based on the cAMP response, 1nM dDAVP was used in the subsequent experiments.

### mpkDCT proteome analysis

**Figure 2:**
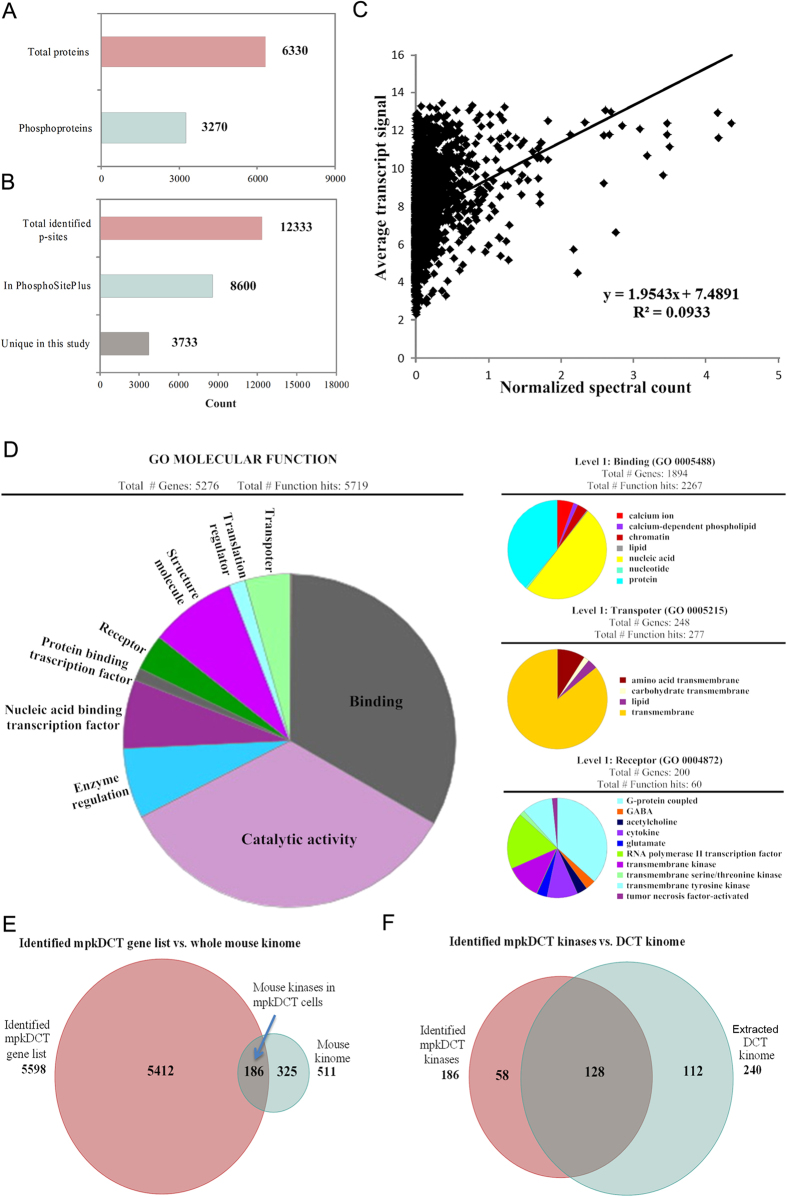
Systems level analysis of mpkDCT cells. (**A**) Total proteins or phosphoproteins identified. Of the 6330 proteins identified, 3270 had at least one phosphorylation site. (**B**) Total phosphosites identified and the number of unique sites not published in the PhosphoSitePlus database. (**C**) Correlation between mpkDCT proteome and a published DCT transcriptome. Normalized spectral count of each protein from the current mpkDCT proteome was plotted against average transcript signal of that particular protein from a published DCT transcriptome^19^. Linear regression was applied and correlation coefficient calculated. (**D**) mpkDCT proteome PANTHER GO-term molecular function analysis. Next level analysis of the molecular function subgroups of binding, transporter activity and receptor activity. (**E**) Venn diagram comparison of current mpkDCT proteome and reported mouse kinome (http://kinase.com/kinbase). (**F**) Comparison of the extracted mpkDCT kinome and kinases identified in a DCT transcriptome^19^.

A SILAC labeling strategy followed by large scale LC-MS/MS based quantitative phosphoproteomics was used to investigate the detailed signaling events in mpkDCT cells following acute dDAVP stimulation. LC-MS/MS analysis of the flow through from the TiO_2_ enrichment step and the eluted phospho-enriched fractions allowed generation of the first in depth mpkDCT proteome and phosphoproteome. A schematic workflow is detailed in Supplemental Fig. 1. In total 6330 proteins were identified with high confidence from mpkDCT cells (Supplemental Table S2), among which 3270 were phosphoproteins containing at least one phosphorylation site (Fig. 2A). The proteome database can also be accessed from: http://interpretdb.au.dk/database/mpkDCT/Total_Proteome.html. Low correlation was observed between the mpkDCT proteome and a published DCT transcriptome^19^ (Fig. 2C and Supplemental Table S3). mpkDCT proteome GO-term molecular function analysis (Fig. 2D), performed using Panther^20^, highlighted a number of major processes highly enriched in DCT cells. These major processes include various transmembrane transporter activities, calcium binding or calcium-dependent phospholipid binding activities (indicating calcium signaling networks), activities of various G-protein coupled receptors, as well as transmembrane receptor protein kinase activities. For example, 75 proteins were identified with known transporter activity (e.g. *Slc* transporters, ion transporters, drug transporters), 2 adenylate cyclase isoforms, 7 steroid receptors and 24 GPCRs (including the V2R). Interestingly, within the GPCRs there were 11 different olfactory receptors and 2 pheromone receptors identified. Further analysis of the proteome versus published databases^21^ identified multiple E1 and E2 enzymes of the ubiquitin/sumo conjugation cascade, in addition to at least 28 E3 ligases (including cullin-3 and Nedd4) and 9 proteins with known deubiquitylation activity.

Analysis of the total mouse kinome, extracted from Kinbase (http://kinase.com/kinbase), with the mpkDCT proteome allowed generation of a comprehensive DCT kinome (Supplemental Table S4), which will facilitate additional studies into the roles of various kinases in DCT function. In total 186 kinases were identified by LC-MS/MS in mpkDCT cells (Fig. 2E). A large number of the identified kinases were observed in a DCT transcriptome generated from Affymetrix microarray data^19^, with 128 kinases being reported in both studies (Fig. 2F).

Although the DCT and cortical collecting duct (CCD) have some morphological similarities, both segments have unique transport characteristics and can express different transport proteins e.g. NCC in DCT and AQP2 in CCD, or similar proteins e.g. ENaC in both segments. To provide initial insights into the origin of this heterogeneity, an mpkCCD proteome was obtained using a similar experimental set up and compared to the mpkDCT proteome. This mpkCCD proteome (Supplemental Table S5) consisted of 8991 unique proteins, which extends current mpkCCD proteome databases (http://helixweb.nih.gov/ESBL/Database/). BINGO GO term analysis of the mpkCCD and mpkDCT proteomes demonstrated that mpkDCT cells were significantly enriched for specific biological processes, whereas mpkCCD cells display a much broader functional distribution of genes and no specific processes reached a level of significance. mpkDCT unique proteins (identified multiple times at the peptide level) showed highly significant over-representation in G-protein linked signaling pathways, regulation of transporter activity and regulation of calcium ion transport (Supplemental Fig. S2).

### mpkDCT phosphoproteome analysis following acute dDAVP stimulation

LC-MS/MS identified 12333 phosphorylation sites in mpkDCT cells (total from dDAVP-treated or control conditions combined). All data and sites identified are in Supplemental Table S6 or can be accessed via http://interpretdb.au.dk/database/mpkDCT/Phosphoproteome.html. The experimental set up allowed identification of at least 4200 different phosphorylation sites from each replicate. Of the identified sites, 8600 were present in the PhosphoSitePlus® database (www.phosphosite.org), while another 3733 are unique to this study (Fig. 2B). 5191 of the identified phosphorylation sites had a phosphoRS^22^ score above 80. To determine the potential functional significances of the phosphorylation sites, we used CPhos^23^. The majority of sites had a site conservation score greater than 0.9 (Supplemental Table S6) indicating that they were highly conserved sites between species and evolution and suggesting that they have important biological functions.

### Modulation of mpkDCT phosphoproteome by AVP

**Figure 3:**
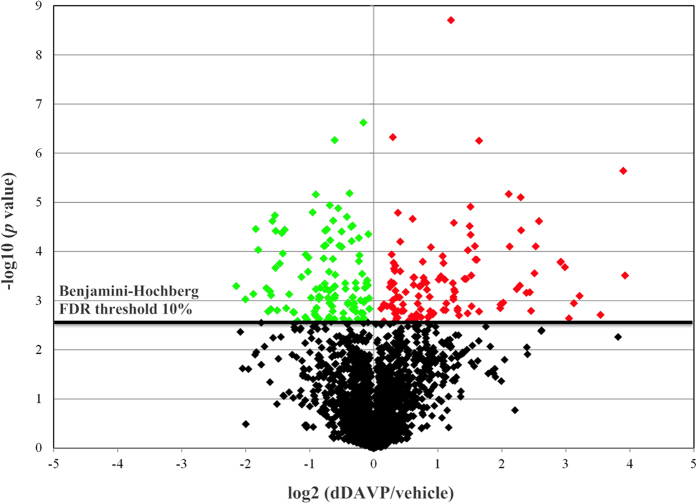
Volcano plot of SILAC-based quantification of phosphopeptides identified between dDAVP or vehicle stimulated mpkDCT cells. Y-axis indicates −log10 (*p* value) while the horizontal axis indicates base 2 logarithmic value of mean peptide abundance ratio (dDAVP vs. vehicle). The horizontal dashed line represents the Benjamini-Hochberg FDR threshold of significance assigned for subsequent analysis of phosphopeptides. Peptides significantly decreased in mpkDCT cells following dDAVP treatment are indicated in green, those significantly increased in abundance are indicated in red.

A Benjamini-Hochberg (BH) FDR threshold of 10% was used for statistical significance and the distribution of phosphopeptides represented as a volcano plot (Fig. 3). In total, we determined with high confidence that 86 phosphorylation sites were increased in abundance following dDAVP stimulation (red points), and 99 sites were decreased in abundance (green points). Final results of up-regulated and down-regulated phosphopeptides (Supplemental Table S6) with their respective phosphorylation sites are accessible at http://interpretdb.au.dk/database/mpkDCT/Phosphoproteome.html.

**Figure 4:**
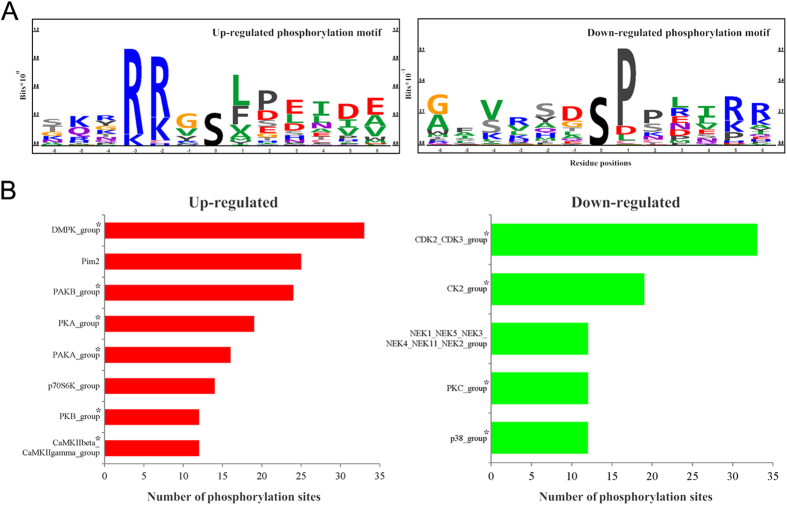
Systems level analysis of phosphorylation in mpkDCT cells. (**A**) Information based sequence logo of the up- and down-regulated phosphorylation motifs following dDAVP stimulation of mpkDCT cells. (**B**) Regulated kinases in mpkDCT cells determined using NetworKIN. The predicted up or down-regulated kinases are shown based on regulated phosphorylation sites. The length of the bars indicates the numbers of predicted phosphorylation sites. *indicates kinase group members identified using LC-MS/MS in this study.

To classify the responsible kinases for the altered phosphorylation sites in a biological context, the amino acid sequences in the proximity of the regulated phosphorylation sites were used for kinase motif prediction using Motif-X^24^. Up-regulated phosphorylation sites were classified into 1 kinase motif: XRXXpSX (score of 16 and fold change of 8.78), consistent with a basophilic phosphorylation motif. Down-regulated sites were classified by 1 proline-directed motif: XXpSPXX (score of 13.36 and fold change of 4.86). To visualize the most dominant positive and negative kinase motifs we used PhosphoLogo^25^. Similarly to Motif-X, following dDAVP stimulation of mpkDCT cells, a basophilic motif was up-regulated and a proline-directed motif was down-regulated (Fig. 4A). Kinase prediction using NetworKIN 2.0^26^ highlighted a number of potential protein kinases responsible for the regulated phosphorylation sites following dDAVP treatment (Fig. 4B). NetworKIN analysis indicated that acute dDAVP stimulation of mpkDCT cells likely increased the activity of kinases in the DMPK, PIM2, PKA, PAKs and CAMK2G families, and decreased the activity of kinases in the CDKs, PKC, and MAPK families, which agrees with the motif prediction analysis. NetworKIN prediction results were mapped against a DCT kinome database^19^ and presented in a form of phylogenetic tree (http://itol.embl.de/ 27), see Supplemental Fig. S3. Despite a limitation of this kinase prediction program (not all kinases are annotated in the NetworKIN database e.g. STK39), important kinase classes potentially involved in dDAVP signaling can be identified based on their evolutionary conservation. For example, the kinases STK39, OSR1 and SGK, which are known to regulate NCC and/or ENaC function, are closely related to particular kinases e.g. PAK and AKT predicted to be responsible for phosphorylation of specific sites following dDAVP treatment.

### Pathway analysis and functional assessment of vasopressin-mediated signaling in mpkDCT cells

**Figure 5:**
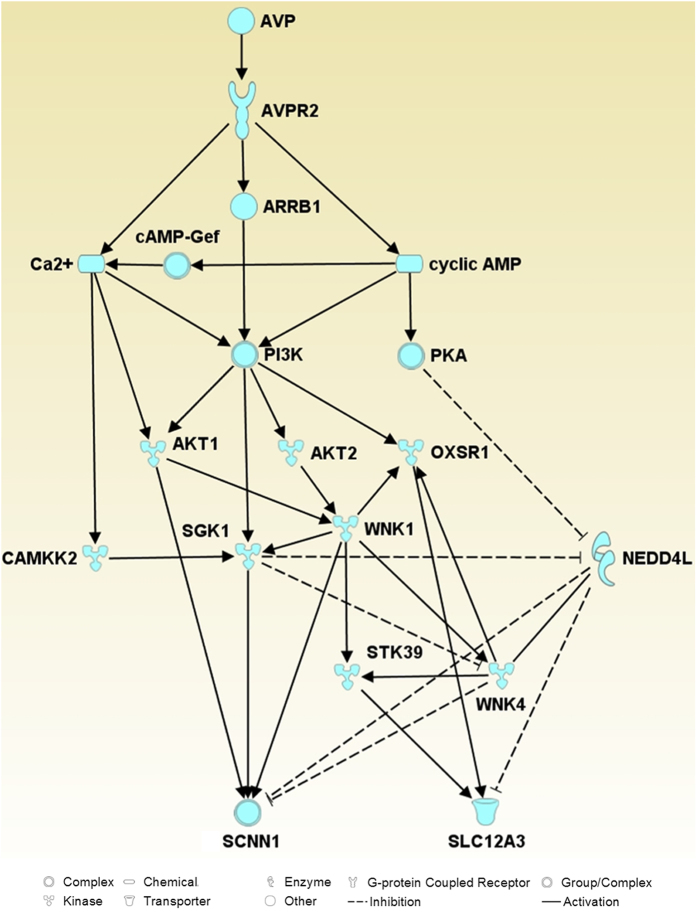
Potential pathways by which ENaC (SCNN1A/B) or NCC (SLC12A3) function can be modulated by vasopressin. Solid arrows indicate activation, dashed lines indicate inhibition. Scheme is a simplified scheme generated using IPA, thus some nodes between proteins have been removed.

Our large-scale data sets (identified phosphoproteins with regulated phosphorylation sites, predicted up or down regulated kinases from NetworKIN analysis) were combined with known modulators of ENaC and NCC (data mined from literature) and IPA core analysis to map and predict relevant VP signaling pathways in mpkDCT cells that may ultimately modulate Na^+^ transport. In accordance with the dDAVP treatment, the top upstream regulator of the pathways was desmopressin (desmopressin acetate or dDAVP) and one of the top canonical pathways identified was Renin-Angiotensin signaling; suggesting that VP-mediated signaling and the renin-angiotensin-aldosterone signaling networks share some similarities. A final complex merged network (interactions and nodes manually checked) is shown in Supplemental Figure S4. A simplified network (Fig. 5) was generated from the complex merged network, which suggests that VP stimulation of mpkDCT cells via the VP type II receptor potentially modulates the function of NCC and ENaC through three major pathways; Ca^2+^, Phosphatidylinositol-4,5-bisphosphate 3-kinase (PI3K) and cAMP. In these networks, CAMKK2, AKT1/2, SGK1 and PKA may be important modulators.

**Figure 6:**
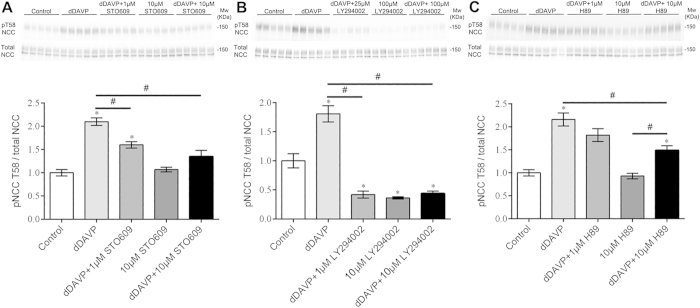
*Ex vivo* studies on isolated cortical tubules from mouse kidney highlight a role for CAMKK and PI3K in VP signaling in mpkDCT cells. Top panels are representative immunoblots and the bottom panels are summarized data. In all experiments, dDAVP (10^−6^ M) treatment for 20 min significantly and consistently increased phosphorylation of NCC at thr-58 (surrogate marker of NCC activation). (**A**) Effects of the CAMKK inhibitor STO609 on baseline and dDAVP stimulated NCC phosphorylation. (**B**) Effects of the PI3K inhibitor LY294002 on baseline and dDAVP stimulated NCC phosphorylation. (**C**) Effects of the PKA inhibitor H89 on baseline and dDAVP stimulated NCC phosphorylation. *represents significant difference from control group, #indicates significant difference between groups as indicated.

To determine a role of PI3K, CAMKK2, PKA or AKT1/2 in the VP-mediated signaling network, *ex vivo* studies on isolated cortical tubules from mouse kidney were performed. These tubule suspensions contain the DCT segment, but also other tubule segments including proximal tubules, CCDs, and some cortical thick ascending limbs. dDAVP (10^−6^ M) significantly and consistently increased phosphorylation of NCC at thr-58 (pT58-NCC, a surrogate marker of NCC activation) after 20 min of stimulation (Fig. 6). The CAMKK selective inhibitor STO609 at 1 μM concentration (K_i_ = 0.5 μM) significantly reduced the dDAVP induced NCC phosphorylation, but basal non-stimulated NCC phosphorylation (10 μM STO609) was not significantly affected (Fig. 6A). In contrast, the PI3K inhibitor LY294002 at 1 μM (K_i_ = 1–3 μM) significantly reduced dDAVP stimulated and, at 10μM concentration, basal pT58-NCC levels (Fig. 6B). H89 (10 μM) had no effect on basal NCC phosphorylation, but small effects on dDAVP-stimulated pT58-NCC levels (Fig. 6C). An AKT1/2 inhibitor (Akt inhibitor VIII, K_i_ = 0.06–0.2 μM) had no significant effect at 1 μM on NCC phosphorylation (data not shown).

## Discussion

The kidney distal convoluted tubule (DCT) plays an essential role in sodium chloride reabsorption, potassium secretion, and magnesium and calcium homeostasis. Due to the small size of the DCT (approx. 1–5 mm in mouse/humans) and difficulty in isolating this segment from surrounding tubules, detailed molecular analysis of the DCT has been hampered. As a basis for understanding the regulatory pathways underlying various homeostatic mechanisms within the DCT, this study utilized large-scale quantitative LC-MS/MS to generate an extensive proteome of a DCT cell line originating from manually dissected mouse DCTs (mpkDCT cells), and assessed the response of these cells to short-term V2 receptor stimulation using phosphoproteomics. Similar to native DCT cells, mpkDCT cells have a gene specific molecular signature that is responsible for providing its specific transport characteristics, including players in transcellular Na^+^, K^+^ and Ca^2+^ transport. mpkDCT cells responded to V2 receptor stimulation with increased intracellular cAMP. Although early studies^28^ using microdissected DCTs suggested that the mouse DCT does not respond to VP, the studies presented here challenge this and are in line with recent studies in mice and rats demonstrating both expression of the V2R in the DCT and a response of this segment to VP stimulation *in vivo*^7^,^11^,^12^,^14^.

Using high sensitivity LC-MS/MS we determined the first comprehensive proteome of mpkDCT cells that will greatly facilitate further studies of DCT function. Little correlation existed between the mpkDCT proteome and a DCT transcriptome^19^, confirming that in the majority of cases e.g.^29^ quantification of mRNA levels is not a good indicator of total cellular protein abundance. 6330 unique proteins were identified in mpkDCT cells, representing a multitude of protein classes with a variety of cellular functions. mpkDCT cells were abundant in proteins involved in cell signaling and molecular transport, including membrane proteins involved in NaCl reabsorption and potassium secretion (e.g. alpha ENaC, Na-K-ATPase, KCC1, KCC4, ROMK, Barttin, BK channels), and calcium and magnesium homeostasis (e.g. TRPV4, Klotho, NCX1, TRPM6). Although NCC mRNA was observed in mpkDCT cells, we did not identify NCC using LC-MS/MS with our data dependent acquisition (DDA) method. Proteins known to play a role in regulation of NaCl reabsorption via NCC or ENaC were also identified, including (but not limited to); the serine/threonine protein kinases WNK1, WNK4, OSR and SPAK; the serine/threonine protein phosphatases 1 and 4 (catalytic and regulatory subunits); the protein phosphatase 1 regulatory subunit 1A (I-1); the E3 ligases Nedd4 and Nedd8; the cullin RING ligase member cullin 3 (CUL3); the calcium-binding protein 39 (Cab39/MO25); and the corticoid oxidative enzyme 11βHSD2. Furthermore, 186 protein kinases were identified in mpkDCT cells, highlighting the complexity of signaling networks in these cells, and allowing investigation of the detailed roles of these kinase families in the DCT for regulating various homeostatic mechanisms. Combined, our data indicate that *in vivo*, DCT cells are likely to be highly specialized cells that are ideally suited to respond quickly to small changes in the external environment via alterations in their transcellular transport capacity.

Of the 6330 proteins identified, 3270 were phosphorylated on at least one site. A total of 12333 phosphorylation sites were identified, with 3733 unique to this study. Two new phosphorylation sites (plus previously known sites) were identified on important proteins for regulating NaCl reabsorption including for example; WNK1 (12 phosphorylation sites, 1 novel), WNK4 (1 site), OSR (3 sites), SPAK (3 sites, 1 novel), and Nedd4L (2 sites). 185 of the detected sites were significantly altered in abundance following acute dDAVP stimulation. The majority of phosphorylation sites increased in abundance with dDAVP were basophilic sites (arginine, lysine, histidine upstream of phosphorylation site), suggesting kinases in the ACG or CAMK families are involved in the downstream signaling mechanisms of VP. In contrast, the majority of decreased sites were proline-directed sites (proline at +1 and −2 surrounding phosphorylation site), indicating that VP-mediated signaling in mpkDCT cells involves down-regulation of CDKs and MAPKs. Similar patterns of kinase up/down-regulation following VP treatment have been observed in collecting duct and thick ascending limb cells^30^,^31^.

Pathway analysis identified various signaling networks activated by VP that may play a role in NaCl transport in the DCT, body sodium balance and ultimately blood pressure. In the following, we briefly discuss the potential pathways for VP effects on modulation of NCC. The actions of VP to increase NCC phosphorylation ultimately depend on SPAK^12^,^14^, but the upstream mediators that modulate SPAK activity following VP treatment are unclear. Data from our *ex vivo* cortical tubule suspensions demonstrate that dDAVP treatment for 20 min significantly and consistently increased phosphorylation of NCC at thr-58. Although the data strongly supports the growing body of evidence that the mouse DCT directly responds to VP, due to the use of a mixed population of tubules in the suspensions, we cannot completely exclude that dDAVP affects another tubule segment and subsequent paracrine signaling modulates NCC function in the DCT. The *ex vivo* studies also suggest that CAMKK and PI3K are important modulators of the effects of VP in the DCT, with PKA possibly playing a minor role. Although to our knowledge the role of CAMKK in modulation of NCC function is novel, the effects of PI3K inhibition on NCC phosphorylation add to the growing body of evidence that under certain conditions e.g. hyperinsulinemic metabolic syndrome, PI3K is an important regulator of NCC activity^32^,^33^,^34^. However, unlike insulin that appears to signal through a PI3K-AKT pathway^32^, inhibition of AKT had no significant effect on dDAVP induced NCC phosphorylation. An alternative pathway through which VP may act is via the serum- and glucocorticoid-inducible kinase 1 (SGK1), an important modulator of sodium reabsorption^35^. The activity of SGK can be modulated by PI3K^35^, CAMKK^36^ and PKA^37^, inhibition of each of these individually in our study reduced NCC phosphorylation to variable degrees. Furthermore, SGK mediated phosphorylation of the E3 ligase Nedd4l decreases its activity, resulting in decreased ubiquitylation and greater cell surface expression of NCC and ENaC (reviewed in^38^). This dual effect of SGK would be predicted to maximally increase sodium transport in the DCT during VP exposure. Further studies to clarify the role of SGK in VP actions on NCC would be informative.

In conclusion, this study provides a comprehensive insight into the proteome and VP regulated phosphoproteome of the DCT. The datasets will facilitate studies into the roles of various kinases, signaling molecules and regulatory proteins for modulation of DCT function. Furthermore, CAMKK and PI3K pathways are central hubs in VP-mediated signaling in the DCT, and under certain pathological conditions, may be important new players for blood pressure regulation.

## Materials and Methods

### Cell culture and SILAC labeling

The mouse kidney distal convoluted tubule cell line (mpkDCT) was cultured as described^39^. Cells were grown at 37 ^o^C/5%CO_2_ in SILAC advanced DMEM/F12-Flex media (Invitrogen) containing 60 nM sodium selenite, 5 μg/ml transferrin, 2 mM glutamine, 50 nM dexamethasone, 1 nM triiodothyronine, 10 ng/ml epidermal growth factor, 5 μg/ml insulin, 20 mM D-glucose and 20 mM HEPES (pH 7.4) in light (contains ^12^C6 lysine, ^12^C6 ^14^N4 arginine) or heavy (contains ^13^C6 lysine, ^13^C6 ^15^N4 arginine) conditions for at least 5 passages to allow >96% labeling efficiency as confirmed by MS analysis. For all experiments, cells were grown on a semi-permeable support (Transwell, Corning) until a fully confluent polarized monolayer was formed (transepithelial resistance (TER) >5 kΩ.cm^2^). On the day of the experiment, cells were incubated in serum-free media for 4 hrs, before the addition of 1 nM dDAVP (Sigma) in serum-free media to the basolateral side of the treated group. Control cells received an equivalent volume of serum-free media. After 15 min cells were washed twice in ice-cold PBS and scraped in cell lysis buffer (8 M urea, 2 M thiourea, 50 mM Tris, pH 7.5) containing protease and phosphatase inhibitors (Halt protease and phosphatase inhibitors, Pierce). After 20 min incubation, lysates were briefly sonicated on ice and centrifuged at 16,000 g for 10 min at 4 ^o^C. Control and hormone-treated samples were pooled in pairs. Pooled samples were reduced, alkylated, and digested using trypsin and desalted using C18 columns (Waters) prior to further fractionation as previously described^40^. A similar approach was utilized for the mouse kidney cortical collecting duct (mpkCCD) cell line^41^.

### Strong cation exchange (SCX) fractionation

Performed by Liquid-Chromatography (Ultimate 3000, Dionex). Peptides were separated on a PolySulfoethyl A SCX column (4.6 mm ID × 20 cm length, 5-μm particle size, 300-Å pore size; PolyLC) at a flow rate of 0.5 ml/min. Buffer A was 5 mM KH_2_PO_4_/25% ACN, pH 2.67. Buffer B was 5 mM KH_2_PO_4_, 500 mM KCl/25% ACN, pH 2.67. A buffer B gradient of 1% to 20% over 30 min followed by 20% to 100% over 5 min was used to separate 1–1.5 mg total peptide mixtures. Fractions were collected every 3 min, vacuum-dried to a final volume of 200 μl and then pooled according to the chromatogram. Pooled fractions were desalted using C18 columns (self-packed C18 columns using the C18 Empore disk, 3M).

### Phosphopeptide enrichment using TiO_2_

Performed as previously described^42^, with minor modifications. TiO_2_ columns were prepared using TiO_2_ powder (GL Science). Peptide samples were resuspended in a loading buffer (80% ACN, 5% TFA, 0.1% glycolic acid) and loaded onto a TiO_2_ column. After two washes in 80% ACN/5% TFA, phosphopeptides were eluted using ammonia water pH 10.5, vacuum dried and resuspended in 0.1% formic acid (FA) for MS analysis.

### Nano-liquid chromatography and mass spectrometry (MS) analysis

Analysis was by nano Liquid-Chromatography (nLC) (Ultimate 3000, Dionex) coupled to a mass spectrometer (Q Exactive, Thermo Fisher Scientific) through an EASY-Spray nano-electrospray ion source (Thermo Scientific). A pre-column (Acclaim®PepMap 100, 75 μm x 2 cm, nanoviper fitting, C18, 3 μm, 100 Å, Thermo Scientific) and analytical column (EASY-Spray Column, PepMap, 75 μm × 15 cm, nanoviper fitting, C18, 3 μm, 100 Å, Thermo Scientific) were used to trap and separate peptides, respectively. For nLC separation, buffer A was 0.1% FA and buffer B was 95% ACN/0.1% FA. A 30 minute gradient of 1% to 35% buffer B was used for peptide separation. Mass spectrometry constituted of full scans (m/z 300–1800) at a resolution of 70,000 (at m/z 200) followed by up to 10 data dependent MS/MS scans at a resolution of 17,500. HCD collision energy was 28%. Dynamic exclusion of 30 s as well as rejection of precursor ions with charge state +1 and above +8 were employed.

### MS data analysis and data inclusion criteria

Raw files were searched against a mouse protein database (RefSeq database downloaded Oct. 2014 containing 58513 sequences) using the SEQUEST algorithm embedded in Proteome Discoverer (PD) software (Thermo Scientific). Quantification was carried out by PD. Precursor mass tolerance was set as 10 ppm and fragment mass tolerance was set as 0.02 Da. Number of maximum miss cleavage sites was set to 2. Carbamidomethylation of cysteine was set as static modification. N-terminal acetylation, methionine oxidation, 10^+^ for heavy arginine, 6^+^ for heavy lysine, as well as phosphorylation of serine, threonine and tyrosine were included as variable modifications. False discovery rate (FDR) was calculated using Percolator and phosphorylation site probability score was evaluated using the phosphoRS 3.0 algorithm^22^. Only rank 1 and high confidence (with a target false discovery rate (FDR) q-value below 0.01) peptides were included in the final results. The quantification of each unique peptide was obtained from the sum of raw values from different peptide charge states and from different SCX fractions. The heavy-to-light ratio was calculated from the summed raw quantification values of the same peptides from both channels. Quantification ratios were then normalized based on median log2 ratio of each biological replicate prior to further analysis. Normalization was carried out on all peptides, including peptides identified from the non-phosphorylated fractions. Peptides identified and quantified in at least three replicates were subjected to Benjamini-Hochberg (BH) FDR estimations, and those that passed the 10% BH-FDR threshold were retained for further analysis.

### Gene Ontology (GO)-term analysis

Performed using Cytoscape version 3.2.0 with BiNGO plugin version 3.0.2, with the combined proteomic data from mpkDCT and mpkCCD cells used as the reference/background set. A hypergeometric test was carried out for overrepresentation analysis. A BH score of 0.01 was used as the significance level.

### Ingenuity Pathway Analysis (IPA)

Core pathway analysis was carried out using a combined data set containing proteins with regulated phosphorylation sites, predicted kinases as well as membrane proteins functionally important in the DCT. Ingenuity knowledge database (gene only) was used as the reference set for analysis. Biologically relevant networks were converged and manually edited based on literature searches.

### Reverse-transcriptase PCR (RT-PCR)

RNA purification and RT-PCR was performed on 2 μg RNA as previously described^43^. RNA extracted from mouse kidney cortex was used as a positive control. PCR primers are in Supplemental Table S1.

### Immunoblotting

Preparation of samples and immunoblotting were as previously described^6^. Primary antibodies were rabbit polyclonal antibodies against total NCC (SPC-402D, StressMarq), NCC phosphorylated at Thr58 (pT58-NCC,^12^), AQP-2^44^, the sodium potassium chloride cotransporter NKCC2^45^, alpha ENaC^46^, gamma ENaC^47^, and the STE20 (sterile 20)-like kinase SPAK^48^, and a mouse monoclonal antibody against parvalbumin (Pvalb, PV235, Swant).

### cAMP assay

Cells were stimulated for 15 min with dDAVP (1 nM or 1 μM, Sigma) or 25 μM forskolin (Sigma) dissolved in DMSO in the presence of 3-isobutyl-1-methylxanthine (IBMX). Controls were DMSO + IBMX. Total cAMP levels were measured using a commercially available cAMP enzyme immunoassay kit (Sigma).

### Mouse cortical tubule suspensions (including ethical approval)

All animal protocols comply with the European Community guidelines for the use of experimental animals, were approved and performed under a license issued for the use of experimental animals by the Danish Ministry of Justice (Dyreforsøgstilsynet) and methods performed in accordance with local guidelines and regulations. For all studies, mice were killed by cervical dislocation. Kidneys from a male c57/bl6 mouse were removed, cortex was dissected into ∼1-mm pieces and placed into 4 ml of enzyme solution containing 1.5 mg/ml collagenase type B (Roche) in buffer B (125 mM NaCl, 30 mM glucose, 0.4 mM KH_2_PO_4_ 1.6 mM K_2_HPO_4_, 1 mM MgSO_4_, 10 mM Na-acetate, 1 mM α-ketogluterate, 1.3 mM Ca-gluconate, 5 mM glycine, 48 μg/mL trypsin inhibitor, and 50 μg/mL DNase, pH 7.4). Samples were mixed continuously at 37 °C and 850 rpm. After 15 min, 2 ml of the enzyme solution was removed and replaced with 2 ml of buffer B. After incubation for 10 min, an additional 2 ml of buffer B was added and the samples incubated for an additional 10 min. Large fragments were allowed to settle, the supernatant removed and centrifuged at 200 × *g* for 2 min. The pellet was resuspended in 5 ml of modified culture medium (HamF12/DMEM containing 0.25 mg/ml DNase, 1 mM HEPES and 30 mM glucose), mixed via pipetting, and centrifuged at 200 × *g* for 2 min. The tubular suspensions were resuspended in 30 ml of modified culture medium and 500 μl transferred into individual tubes containing either DMSO (control), STO609, H89 or LY294002 (Sigma). Suspensions were incubated with continuous mixing for 15 min at 37 °C, 850 rpm. Where indicated, dDAVP (1 μM) was added and suspensions incubated for an additional 20 min. Tubules were centrifuged for 10 min at 3,000 × *g* at 4 °C, pellets resuspended in 200 μl Laemmli sample buffer containing DTT (50 mg/mL) before immunoblotting.

### Statistics

Benjamini-Hochberg (BH) FDR estimation was used for statistical evaluation of the MS derived ratios of unique peptides between biological replicates. A BH FDR threshold of 10% was used as the confidence cutoff. For data derived from cAMP assays or *ex-vivo* tubule suspensions, data fitting a normal distribution were analyzed using one-way ANOVA followed by Tukey’s Multiple Comparison Test. Multiple comparison tests were applied only when a significant difference (*p* < 0.05) was determined in the ANOVA. All data are presented as mean ± s.e.m.

## Additional Information

**Accession Codes**: The mass spectrometry proteomics data for mpkDCT have been deposited to the ProteomeXchange Consortium^49^ via the PRIDE partner repository with the dataset identifier PXD001729.

**How to cite this article**: Cheng, L. *et al.* A Systems Level Analysis of Vasopressin-mediated Signaling Networks in Kidney Distal Convoluted Tubule Cells. *Sci. Rep.* **5**, 12829; doi: 10.1038/srep12829 (2015).

## Supplementary Material

Supplementary Information

Supplementary Information

Supplementary Information

Supplementary Information

Supplementary Information

Supplementary Information
