# Supplementary material for: A Systems Level Analysis of Vasopressin-mediated Signaling Networks in Kidney Distal Convoluted Tubule Cells
Source: Sci Rep. 2015 Aug 4;5:12829. doi: 10.1038/srep12829 (PMC4523861; doi:10.1038/srep12829)
Supplement: Supplementary Information [file srep12829-s1.pdf]

Supplemental Information for

A Systems Level Analysis of Vasopressin-mediated Signaling Networks in Kidney Distal Convoluted Tubule Cells

Lei Cheng[1‡], Qi Wu[1‡], Marleen L. A. Kortenoeven[1], Trairak Pisitkun[1,2‡] and Robert A. Fenton[1‡*]

[1]Center for Interactions of Proteins in Epithelial Transport, Department of Biomedicine, Aarhus University, 8000, Aarhus, Denmark

[2]Faculty of Medicine, Chulalongkorn University, Bangkok, Thailand

[‡]These authors contributed equally

Supplemental Figure S1. Schematic overview of the experimental workflow. Cells were grown in either SILAC light or heavy media on filter plates until confluent and stimulated with dDAVP (1nM) for 15 min from the basolateral side. Samples were pooled for further proteomics analysis and quantification. Peptide quantification was performed at the MS1 level. Four biological replicates were performed on different days.

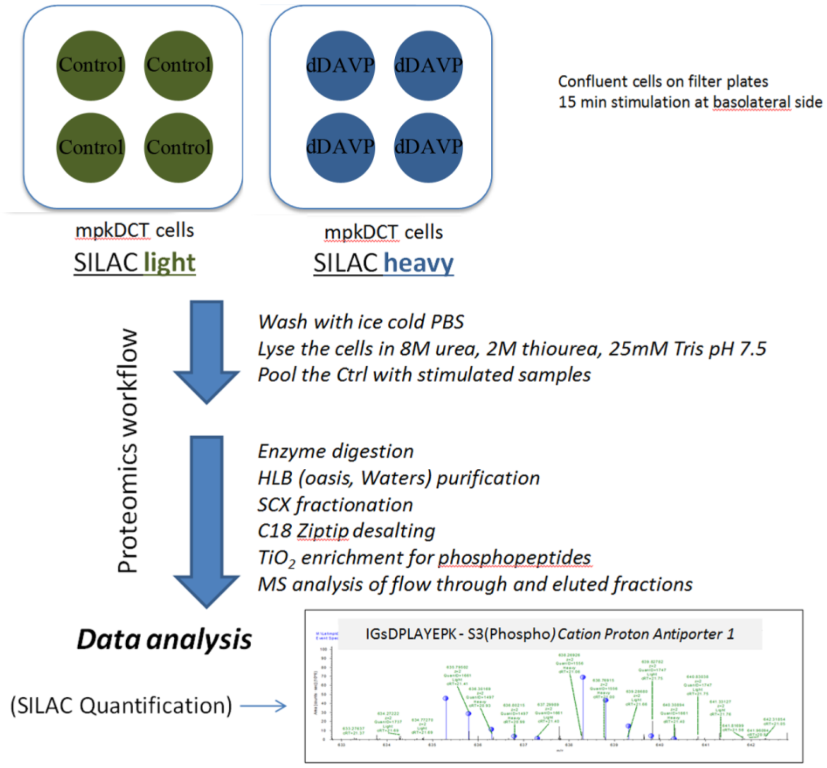

Supplemental Figure S2. mpkDCT cells are over-represented in proteins involved in signal transduction, cell surface receptor linked signaling and G-protein linked signaling pathways. GO Biological Process analysis was performed using Cytoscape version 3.2.0 with BiNGO. Background utilized was a combination of mpkDCT and mpkCCD genes. Yellow nodes represent GO categories that are overrepresented at the significance level (p<0.01). For more significant p-values, the node color gets increasingly more orange. Uncolored nodes are not overrepresented GO categories, but are the parents of overrepresented categories further down the tree.

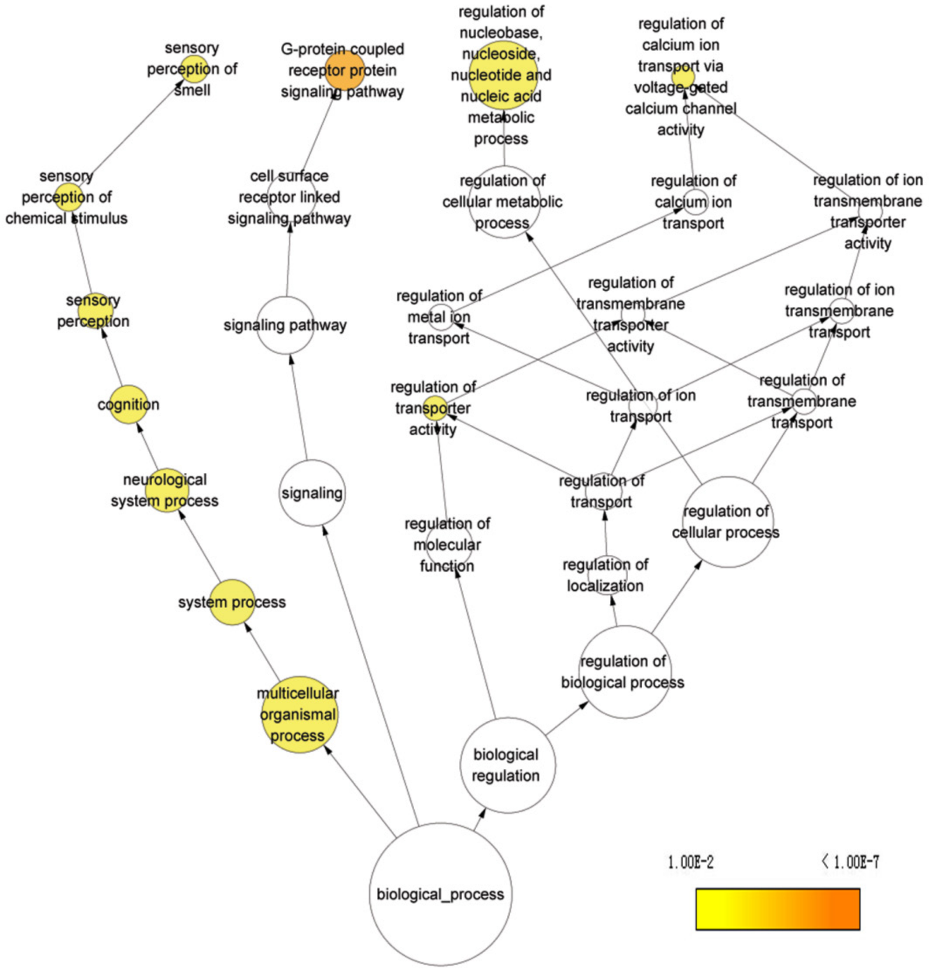

Supplemental Figure S3. A kinase dendrogram generated from DCT kinase transcriptome data (Pradervand, S., Zuber Mercier, A., Centeno, G., Bonny, O., and Firsov, D. (2010) A comprehensive analysis of gene expression profiles in distal parts of the mouse renal tubule. Pflugers Archiv : European journal of physiology 460, 925-952), including 240 Ser/Thr kinases that had transcript signal intensities above background). Red and green bars indicate numbers of the up- and down-regulated phosphopeptides predicted to be phosphorylated by a given kinase based on the NetworKIN algorithm.

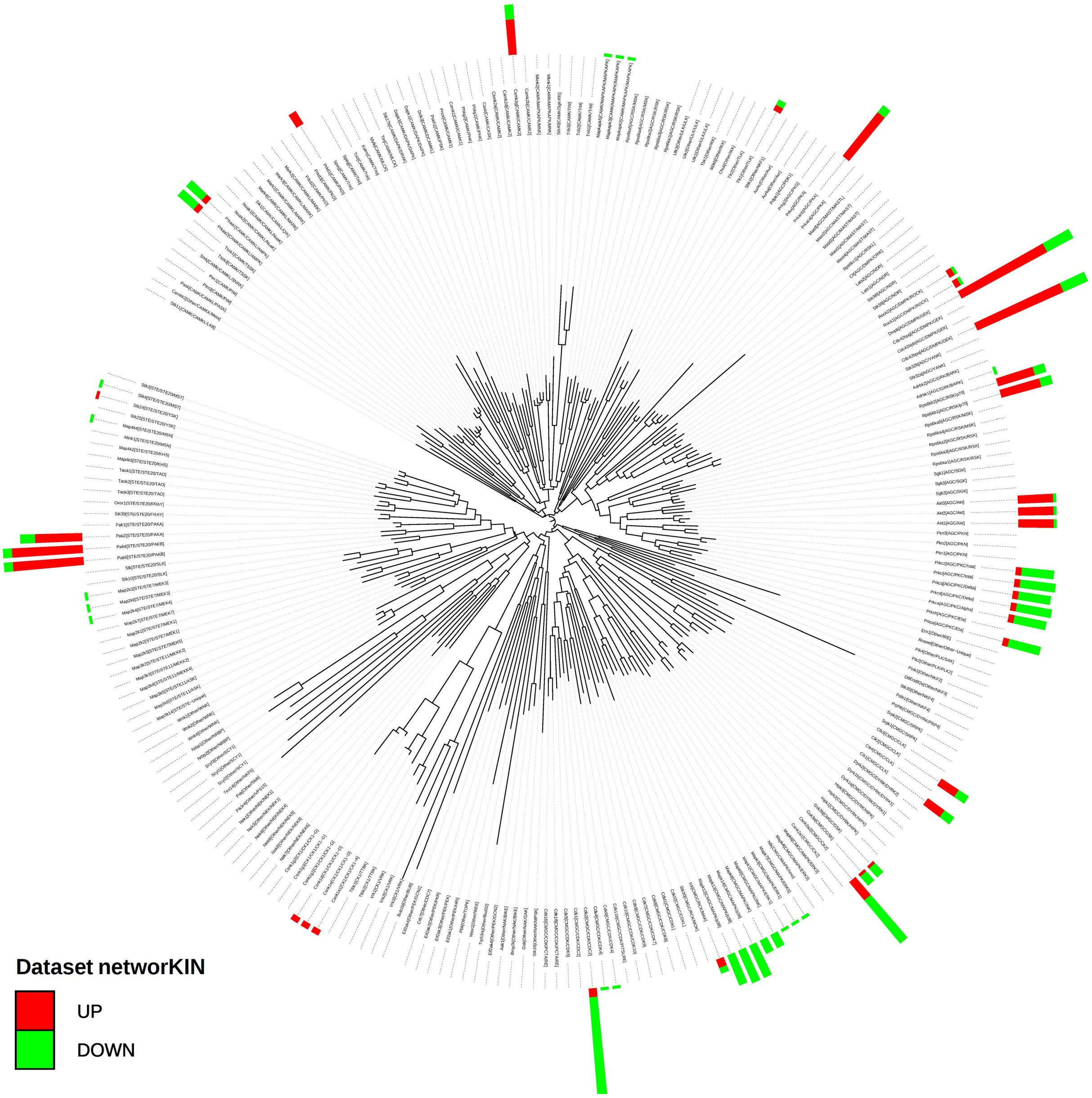

Supplemental Figure S4. Pathway analysis of vasopressin signaling events in mpkDCT cells. Complex pathway is generated using IPA and has been manually edited for confirmation. Dashed lines with arrows indicate activation pathways, solid lines indicate protein:protein interactions, lines ending with bar indicate inhibition. Green nodes are signaling molecules, red nodes are proteins/protein complexes.

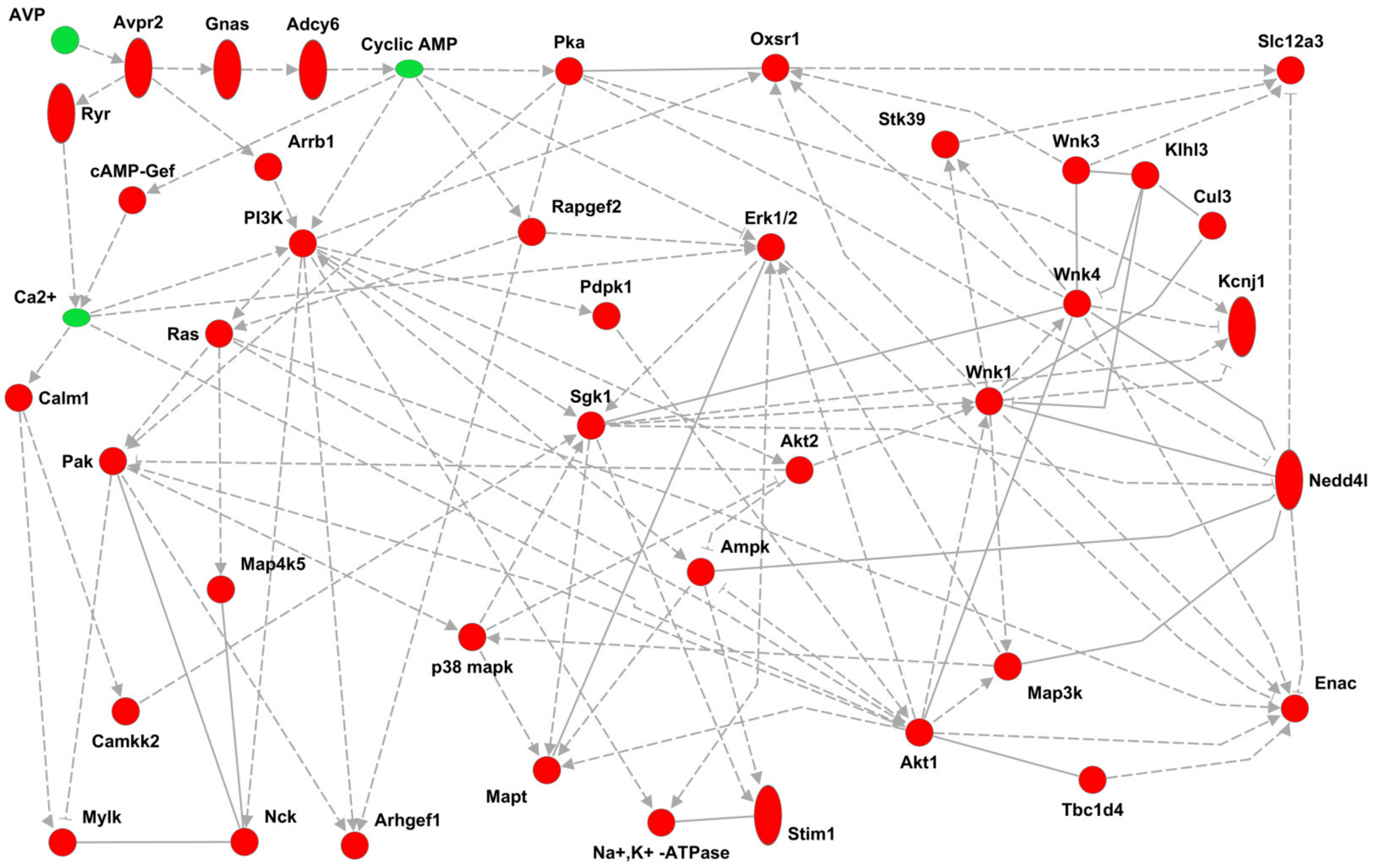

Supplemental Table S1. PCR primers.

Gene target	Forward primer (5'-3')	Reverse primer (5'-3')	Product length (bp)
Actin	ACATGGCATTGTTACCCACTGG	CGGACTCATCGTACTCCTGCTT	956
Aqp2	AACTCCGGTCCATAGCGTTC	GGAAGAGCTCCACAGTCACC	402
Slc12a1	AAGCGGGAATTGGTCTTGGA	CACGGTGATGGAACCGATGA	311
Slc12a3	CCTGCATTCATTCCTCAAGCA	GTAATCCAGGGGAGTCGCAG	225
Scnn1a	TTGACGTCTCCAACTCACCG	CTGGTTGCACAGTTGGAAGC	728
Scnn1b	CACACTGGAGCAGCTTCCTAA	AAGTTCCGATGACTGCAGGG	812
Scnn1g	CGAAGAACTGCTGGTGACCT	TTGCTTGCACACTGATTGGC	607
Trpm6	CCGGGTTCTTTCCTGACTCC	TATGGCCGAGAGGTCCTGAA	531
Pvalb	ACGGCAAGATTGGGGTTGAA	CTTGCCAAACCAACACCCTG	286
Avpr2	GTGTCTACCACGTCTGCAGTG	GGTCTCGGTCATCCAGTAGC	91
Nr3c2	TGAGTTCCTTTCCGCCTGTC	GCCTCATCTCCACACACCAA	190
CaBP-D28k	AACTGACAGAGATGGCCAGGTTA	TGAACTCTTTCCCACACATTTTGAT	87
Agtr1a	GTTGGAACCTGCGGAGTAGC	GTCAGCCAGGGCGAGATTTA	572
Stk39	TTCAAGTGGAAGGTCGTCCG	TACGTTGGGATGGCTGCATT	457
Oxsr1	GTGCCCCCAAAAAGGAGAGA	TGTAGCAATGGTTGGCTCGT	269
